# The oxytocin system promotes resilience to the effects of neonatal isolation on adult social attachment in female prairie voles

**DOI:** 10.1038/tp.2015.73

**Published:** 2015-07-21

**Authors:** C E Barrett, S E Arambula, L J Young

**Affiliations:** 1 Center for Translational Social Neuroscience, Silvio O. Conte Center for Oxytocin and Social Cognition, Division of Behavioral Neuroscience and Psychiatric Disorders, Yerkes National Primate Research Center, Emory University, Atlanta, GA, USA; 2 Department of Psychiatry and Behavioral Sciences, Emory University, Atlanta, GA, USA

## Abstract

Genes and social experiences interact to create variation in social behavior and vulnerability to develop disorders of the social domain. Socially monogamous prairie voles display remarkable diversity in neuropeptide receptor systems and social behavior. Here, we examine the interaction of early-life adversity and brain oxytocin receptor (OTR) density on adult social attachment in female prairie voles. First, pups were isolated for 3 h per day, or unmanipulated, from postnatal day 1–14. Adult subjects were tested on the partner preference (PP) test to assess social attachment and OTR density in the brain was quantified. Neonatal social isolation impaired female PP formation, without affecting OTR density. Accumbal OTR density was, however, positively correlated with the percent of time spent huddling with the partner in neonatally isolated females. Females with high accumbal OTR binding were resilient to neonatal isolation. These results are consistent with the hypothesis that parental nurturing shapes neural systems underlying social relationships by enhancing striatal OTR signaling. Thus, we next determined whether early touch, mimicking parental licking and grooming, stimulates hypothalamic OT neuron activity. Tactile stimulation induced immediate-early gene activity in OT neurons in neonates. Finally, we investigated whether pharmacologically potentiating OT release using a melanocortin 3/4 agonist, melanotan-II (10 mg kg^−1^ subcutaneously), would mitigate the social isolation-induced impairments in attachment behavior. Neonatal melanotan-II administration buffered against the effects of early isolation on partner preference formation. Thus, variation in accumbal OTR density and early OT release induced by parental nurturing may moderate susceptibility to early adverse experiences, including neglect.

## Introduction

Offspring who have experienced disruptions in parental nurturing often display increased fear responsiveness, hyperactive stress physiology, impaired social competence, and, in humans, an increased vulnerability for mood and anxiety, addiction and personality disorders.^1, 2, 3, 4, 5^ The quality of early parental care is a salient predictor of adult socioemotional behavior and neurobiology.^6, 7, 8^ Supplemental tactile stimulation to simulate parental nurturing alleviates some deficits resulting from maternal separation in both rats and voles.^9, 10^ In humans, interventions that apply supplemental touch to preterm infants improve emotional self-regulation and social reciprocity throughout development.^11, 12, 13^ One candidate mechanism by which early tactile stimulation modulates behavior is oxytocin (OT) signaling.^14, 15^ OT is released centrally and peripherally after touch in adult rats,^16, 17, 18^ and is increased in the saliva of human infants after parental interactions.^19, 20^ Elevated licking and grooming and peripheral OT injections in rat pups similarly enhance adult maternal behavior.^21, 22^ Early care shapes OT neural pathways, and rat offspring exhibit an oxytocin receptor (OTR) profile characteristic to that of their rearing mother.^23, 24^ Together these findings suggest that early OT signaling mediates some of the behavioral changes induced by variation in parental nurturing.

The prairie vole (*Microtus ochrogaster*) provides an excellent opportunity to assess the impact of parental nurturing on offspring neural and social behavioral development.^25, 26^ Remarkable individual variation in neural OTR and vasopressin 1a receptor expression has been associated with variation in alloparental and pair bonding behaviors in outbred laboratory colonies.^27, 28, 29, 30, 31, 32, 33, 34, 35^ In adult female prairie voles, OT signaling in the nucleus accumbens (NAcc) facilitates pair bond formation as determined by assessing partner preferences.^30, 36, 37^ Furthermore, manipulating accumbal OTR density at weaning alters adult alloparental care and bonding in prairie voles.^29, 38^ Early experience impacts neuropeptide circuitry underlying social attachment in both rats and voles.^39, 40, 41, 42, 43^ In the socially monogamous mandarin vole, early social deprivation impairs adult social bonding, reduces social interactions and enhances anxiety-like behavior in adulthood, and some of these effects are reversed with supplemental tactile stimulation.^44, 45^

Here, we hypothesized that early-life social experiences impact adult social attachment in prairie voles by assessing partner preference formation, and that responses to early experiences (for example, nurturing or neglect) are, in part, mediated by early OT signaling. In Experiment 1, we investigated the effect of repeated neonatal social isolations on adult social attachment and OTR receptor density, as well as anxiety-related behavior in the open field, which may contribute to social variation. We hypothesized that this early social isolation would impair adult bonding and that variation in OTR binding would generate diversity in the degree an individual was affected. As polymorphic variation in human *OXTR* gene has been associated with susceptibility to early adverse experiences^46, 47, 48, 49^ and NAcc OTR density is highly variable and correlated with alloparental care and female pair bonding,^30, 34^ we *a priori* predicted that accumbal OTR density would be correlated with partner preference (PP) behavior in an experience-dependent manner. We, therefore, examined the interaction between OTR binding and early experience on partner preference in the females from Experiment 1 after the final behavioral testing.

Parental nurturing alters physiology, neurobiology and behavior, and licking and grooming is an important component of parental care.^6^ Tactile stimulation may functionally translate into neural changes via activation of central neuropeptide systems.^17, 50^ In Experiment 1, females with high OTR binding may have experienced heightened social buffering in response to an observed burst in licking and grooming upon reunion to the nest. In Experiment 2, we tested the hypothesis that neonatal touch, simulating parental licking and grooming, activates OT neurons in 7-day-old pups by analyzing the expression of the immediate-early gene early growth response protein-1 (EGR-1), which is a robust marker of hypothalamic neural activity.^51^ Pups were euthanized after 5 min of paint brush stimulation and EGR-1 immunoreactivity in OT, as well as vasopressin (AVP) neurons was quantified.

As females with high striatal OTR binding displayed partner preferences despite exposure to neonatal social isolations, we hypothesized that enhancing neonatal OT release would recapitulate this potential mechanism of resiliency by enhancing OTR signaling during social isolation. Given that OT does not efficiently cross the blood–brain barrier and daily central OT injections would be highly stressful, we targeted central OT neurons with a melanotan-II (MTII), a melanocortin (MC) 3/4 receptor agonist. A peripheral injection of MTII potentiates OT release in the prairie vole NAcc and facilitates adult PP formation through an OTR-dependent mechanism.^52^ The endogenous MC, α-melanocyte stimulating hormone, stimulates central OT release in rat hypothalamic slices via the MC4 receptor.^52, 53^ Recently, we found that MTII stimulates immediate-early gene activity in OT neurons of week-old prairie vole pups and treatment with MTII for the first week of life facilitates adult partner preference in female prairie voles.^54^ In Experiment 3, we hypothesized that neonatal treatment with MTII, which would potentiate OT release in response to endogenous stimulation (for example, tactile stimulation), would enhance social buffering to neonatal social isolations and rescue impairments in PP formation. Therefore, we administered MTII during the first week of the 2-week social isolation paradigm and examined the impact on adult PP formation.

Together, our results suggest that neonatal OTR signaling, in response to parental tactile stimulation, may positively influence the development of neural systems involved in adult social attachment. Furthermore, variation in neural sensitivity to OT in the NAcc predicted susceptibility and resilience to bouts of neonatal social isolation with respect to later-life social behaviors. Finally, augmenting OT signaling may mitigate the deleterious effects of neonatal isolation on adult social attachment. These results are consistent with the observation that genetic variation in the human *OXTR* gene is associated with differential susceptibility to the negative psychiatric outcomes of early adversity in humans,^46, 47, 48^ as well as endocrine studies showing that parental engagement leads to increased OT release in infants.^19, 20^

## Materials and methods

### Animal care and handling

**Figure 1:**
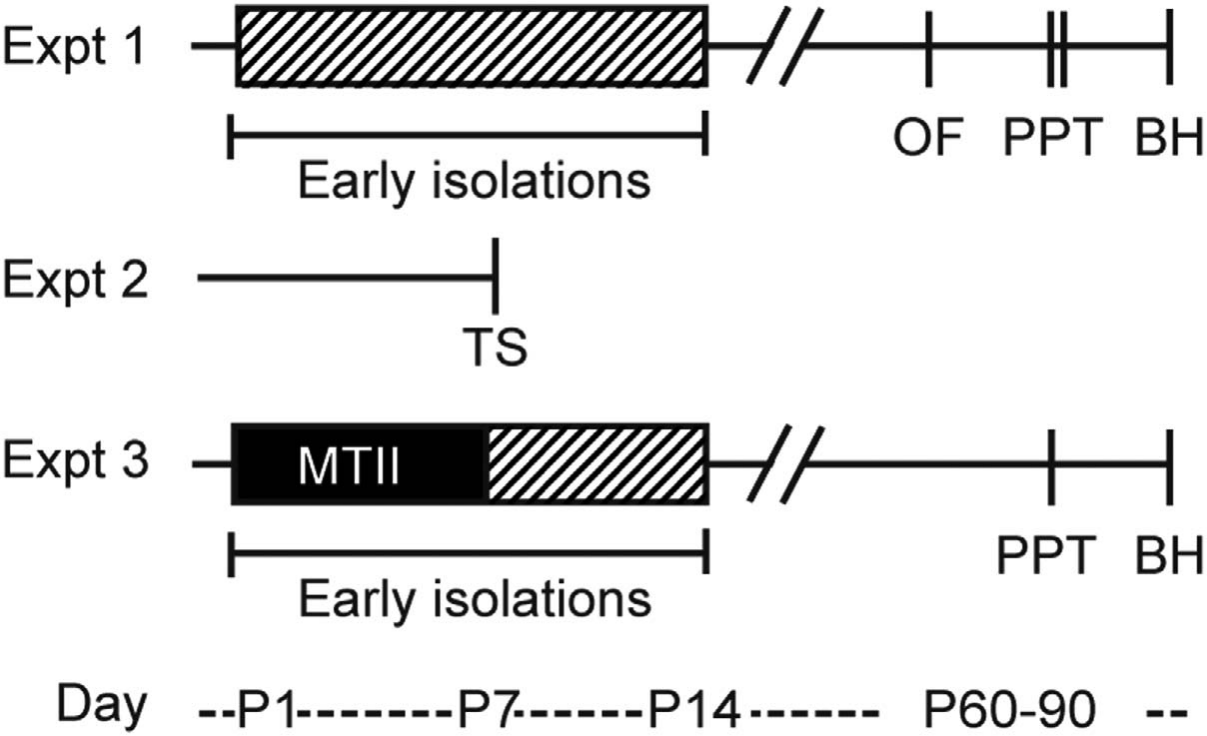
Experimental design. In Experiment (Expt) 1, entire litters were isolated from both parents and siblings in a temperature-controlled incubator for 3 h per day from PND1–14, with PND0 as the day of birth. Control litters were left undisturbed, apart from being weighed every 3 days. Anxiety-like behavior was tested in the open field (OF), social attachment in the partner preference test (PPT) after 24 and 48 h of cohabitation, and brains were harvested (BH) to perform autoradiography for OTR. Brains from a subset of females who were naive to behavioral testing also underwent autoradiography. In Expt 2, the impact of a 5min body and anogenital paint-brush tactile stimulation (TS) on hypothalamic oxytocin (OT) and vasopressin (AVP) neuronal activation was tested in PND6–7 pups. For 1 h before and after stimulation, pups were kept in a temperature-controlled incubator. In Expt 3, pups were isolated as in Expt 1, but given melanotan-II (MTII) or saline daily for the first week of life. OTR, oxytocin receptor; PND, postnatal day.

Experimental timelines are outlined in Figure 1. Outbred prairie voles, originally descended from a wild-caught population in Illinois, USA, were maintained on a 14:10 h light:dark cycle at 22 °C with access to food (Purina high-fiber rabbit chow) and water *ad libitum*. Breeder housing consisted of ventilated cages (34 × 30 × 19 cm) lined with bedding (Bed-o'Cobs, Andersons Lab Bedding, Maumee, OH, USA). Cages were not changed for the first three postnatal days (PNDs). Female subjects were weaned (PND21) into same-sex same-treatment pairs or trios in cages (30 × 18 × 19 cm) before the birth of the next litter. No more than two animals per sex and experimental group were used from the same litter within each experiment. Litters ranged between two to three pups, and those with more than six pups were culled. Sample sizes for behavioral and immunohistochemistry experiments were chosen on the basis of previous experiments with similar measurements.^42, 52, 54^ All the procedures were conducted in accordance with the National Institutes of Health Guide for the Care and Use of Laboratory Animals and were approved by the Emory University Institutional Animal Care and Use Committee.

### Experiment 1. Effect of neonatal social isolation on adult behavior and oxytocin receptor binding

#### Procedure

Between PND1–14, whole litters were removed from the cage and isolated in single holding units in a temperature-controlled incubator (30–32 °C) from 0900–1200 h. To assess the impact of isolation on weight, control and experimental litters were weighed every 3 days and at weaning. From PND1–14, weights were averaged within each litter and sexes were not determined until weaning. In this experiment, no more than one animal per sex per litter was used for behavioral analysis or OTR density analysis. Weights were analyzed with repeated measures analysis of variance (ANOVA) with day as a repeated measure and rearing condition as a between-subjects factor followed by a *post hoc* Student's *t*-test when appropriate. Weanling weights were analyzed with Student's *t*-tests between groups.

#### Effect of paradigm on parental care upon reunion

To assess potential parental influences mediating the effects of isolation, parental care upon reunion of pups to the home cage was investigated. In another cohort, home cage observations of parental care were taken between PND1–14 during three 1 h blocks (1200–1300 h, immediately after reunion; 1500–1600 h; 1800–1900 h) similar to previously described protocols.^42^ Twelve breeder pairs were observed (five control, seven early-isolated), and early manipulations were performed as described above. Within each 1 h block, 15 point observations were taken every 4 min. Dams and sires were scored for nest occupancy and type of activity: licking and grooming pups; carrying pups; self-grooming; grooming mate; resting; nest building; wandering around cage; eating; drinking; digging; and climbing. Females were also monitored for active (arched-back or standing crouch) or passive (blanket or side) nursing status. The frequency of each behavior was calculated by dividing by the total number of observations (630 over PND1–14, 210 per each observation block over PND1–14). Repeated measures-ANOVA was performed on behavioral data (licking and grooming, nursing) across time points, and *post hoc t*-tests were performed where appropriate.

#### Open field

To assess the impact of neonatal social isolation on adult anxiety-like behavior, which could affect social behavior, adults (~80 days) were tested for willingness to explore an open field as previously described.^42^ Subjects were placed in a corner of an open-field arena (40 × 40 × 40cm) and the duration and distance moved within the center or periphery was recorded using an automated system (SocialScan 2.0, Clever Sys, Reston, VA, USA).^42^ Open-field data (duration in center, periphery; latency and frequency to enter center; distance traveled in center, periphery, total arena) were analyzed with multivariate ANOVA.

#### Partner preference

The PP test was used to assess the impact of neonatal social isolation on adult bond formation as previously described.^42, 55^ At ~90 days of age, female subjects (*n*=10 control, 10 isolated) were paired in a clean cage with a sexually experienced vasectomized male partner to prevent pregnancy-induced neural changes. Males (*n*=12 control, 10 isolated) were paired with a sexually experienced ovariectomized female partner primed with estradiol benzoate (Sigma-Aldrich, St. Louis, MO, USA; BP958) 3 days before testing to induce sexual receptivity.^28^ Subjects were tested for partner preference after cohabitating for 24 h, placed back in the same cage for a second overnight cohabitation and tested again at 48 h. During the PP test, subjects were allowed to freely roam a three-chambered arena with the partner and a novel stranger male tethered to either end. An overhead camera recorded the 3 h test, and time huddling with either stimulus animal was scored with an automated system (SocialScan 2.0, Clever Sys).^42^ Female prairie voles are induced ovulators and take ~2 days of exposure to male urine to go into estrus and mate,^56^ thus female subjects likely began mating toward the end of the 48 h cohabitation.

Time spent huddling with the partner and stranger animals was analyzed with ANOVAs with rearing condition, stimulus animal (partner or stranger) and sex as between-subjects factors. Planned paired *t*-tests were performed on huddling time with partner versus stranger to assess PP within each group, and corrected for multiple comparisons within each experiment using the Bonferroni–Holm test.

#### Tissue collection and OTR autoradiography

Four days following PP testing, animals were euthanized by CO_2_ asphyxiation in their home cages and brains were collected in isopentane on dry ice. OTR autoradiography was only performed on females, as no behavioral effects were detected in males. In addition to behaviorally experienced female brains, autoradiography was performed on a separate cohort of behaviorally naive female littermates (*n*=5 control, 6 isolated) to determine whether neonatal isolation altered OTR density. Autoradiography for OTR binding was performed with 0.05 nM ^125^I-OVTA (PerkinElmer, NEX 254050UC, Waltham, MA, USA) as previously described.^42, 57^ Specific binding was calculated by subtracting nonspecific background binding in the S1 region of the cortex from total binding in each region. Optical density measures were taken across three sections in the prefrontal cortex, NAcc shell and core, lateral septum, bed nucleus of the stria terminalis and the central and basolateral amygdala.

To determine whether early rearing alters OTR binding, autoradiographic data were analyzed with multivariate ANOVA across brain regions between control and isolated groups. To investigate the relationship between OTR density, rearing condition and behavior, multiple linear regressions were performed with OTR binding and early rearing condition as independent variables and behavioral measures as dependent variables. For partner preference data, percent time huddling with the partner (partner huddling/total huddling time with the stranger and partner × 100) was used as a dependent variable and interaction effects were Bonferroni–Holm corrected for the number of comparisons (*N*=6) across brain regions. Data from the 48 h PP test was used, as control females did not display a partner preference until this time point. Since the regression analysis revealed an interaction between NAcc OTR density, rearing condition and partner preference behavior, we performed a *post hoc* analysis using a median split to categorize animals as having high or low OTR binding in the NAcc. An ANOVA was performed with rearing condition, NAcc OTR (high versus low) and stimulus animal (partner versus stranger) as between-subject factors. Planned paired *t*-tests were performed on huddling time with partner versus stranger to assess PP within each OTR binding group, and corrected for multiple comparisons within each experiment using the Bonferroni–Holm test.

### Experiment 2. Effect of tactile stimulation on immediate-early gene expression in hypothalamic oxytocin and vasopressin neurons

#### Procedure and tissue collection

To test whether simulating parental licking and grooming stimulates OT neurons in prairie vole neonates, we investigated the activity of hypothalamic (paraventricular nucleus, PVN) OT and vasopressin neurons in response to tactile stimulation. Immediate-early genes, such as EGR-1, are widely used as an indicator of neural activation. Prairie vole pups (PND6–7) were removed from the nest and put in individual holding units in a 30 °C incubator. After 1 h, pups were removed by a gloved investigator and stroked (tactile stimulation, TS) with a damp stiff-haired paint brush to mimic licking and grooming^58^ or only held (handled, H) for 5 min to control for any impact of separation and handling alone on neuropeptide systems. A total of 15 pups from five different litters were used in the experiment (*N*=3H females, 4H males, 4TS females, 4TS males), and treatment was randomized within a litter. Tactile stimulation consisted of 3 min of body stimulation and 2 min of anogenital stimulation, alternating every 1 min. After stimulation, subjects were returned to the incubator. Pups were deeply anesthetized with isofluorane and euthanized by rapid decapitation 1 h following the stimulation. Gonads were examined to determine sex. Brains were removed and post-fixed in 10 ml of 5% acrolein diluted in phosphate-buffered saline (pH 7.4) for 3 h. Brains were transferred to 30% sucrose (4 °C) for at least 24 h, sectioned on a microtome at 40 mM and stored in cryoprotectant (4 °C) until immunohistochemical processing.

#### Immunohistochemistry

Every third section was processed for EGR-1 and either OT or AVP, as previously described.^54^ EGR-1 was chosen as a measure of neural activity as it has been reported to be a more sensitive marker of activity in the hypothalamus than Fos.^51^ Briefly, sections were incubated with 1:8000 rabbit polyclonal anti-EGR-1 (sc-189, Santa Cruz Biotechnology, Santa Cruz, CA, USA) and either 1:10 000 mouse monoclonal anti-OT (mAb5296, Millipore, Billerica, MA, USA) or 1:1000 mouse monoclocal anti-VP (PS41, generously donated by Dr H Gainer, NIH, USA^59^). Sections were washed in phosphate-buffered saline and incubated in secondary Alexa Fluor conjugated antibodies for 3 h at 4 °C (1:1000 Alexa Fluor 568 for EGR-1 and Alexa Fluor 488 for OT or AVP, Life Technologies, Grand Island, NY, USA). Tissue was mounted and cover slipped using Vectashield mounting medium with Dapi (Vector, Burlingame, CA, USA) and Z-stack images were taken on a Leica confocal microscope at 40 × magnification. The total number of OT or AVP cells and EGR-1 double-labeled neurons in the PVN were counted in five to six bilateral sections for OT and two to five for AVP from each subject by an experimenter masked to the sex and treatment condition. The total percentage of EGR-1-positive OT or AVP cells was calculated across all sections for each animal, and analyzed with planned Student's *t*-tests between groups.

### Experiment 3. Effect of MTII treatment for the first week of the 2-week neonatal social isolation on adult partner preference

#### Procedure

Whole litters were exposed to early isolation (PND1–14) as described above. Within a litter, individual pups were injected with either MTII (Alpha Diagnostics International, San Antonio, TX, USA) or saline. Pups were toe clipped to identify drug- or saline-treatment groups. Neonates received 28 μg MTII (35 μl) in 0.9% sterile saline (Hospira, Lake Forest, IL, USA) for the first 2 days of life, 40 μg MTII (50 μl) for days 3–5 and 52 μg MTII (65 μl) for days 6–7 or an equivalent volume of saline. These doses are roughly equivalent to the 10 mg kg^−1^ doses given to prairie voles in previous studies.^52, 54^ Weights were measured daily between PND1–14 and on PND21, and analyzed as described above.

#### Partner preference

To determine whether daily MTII treatment enhanced adult bonding, female subjects were tested for their preference for an opposite-sex conspecific after a shortened non-mating cohabitation. For females, a 6 h cohabitation without mating is not normally sufficient to form a preference, and early MTII treatment induces adult partner preference under these conditions.^54^ At ~60 days of age, females were paired with a sexually naive, intact opposite-sex partner. Females (*N*=15 saline, 14 MTII) were paired for 6 h and videotaped to ensure that no mating occurred. One female that mated within the MTII group was excluded from behavioral analyses. PP data were analyzed with ANOVA with drug and stimulus animal as between-subjects factors, and with planned paired *t*-tests with Bonferroni–Holm corrections.

#### Statistical analyses

Data are presented as mean±s.e.m., unless otherwise noted. Data were analyzed for normality with the Kolmogorov–Smirnov test and for equality of variance with Levene's Test for Equality of Variances. Partner preference data from the MTII experiment did not meet criteria for normality, therefore, the ANOVA was performed on log-transformed data followed by nonparametric Wilcoxon sign ranked tests on partner versus stranger huddling. Statistics were performed with SPSS Statistics 17.0 (IBM, Armonk, NY, USA) with significance set at *P*<0.05 and two-tailed tests, unless otherwise specified.

## Results

### Experiment 1. Effects of neonatal social isolation on adult behavior and oxytocin receptor binding

#### Weight

**Figure 2:**
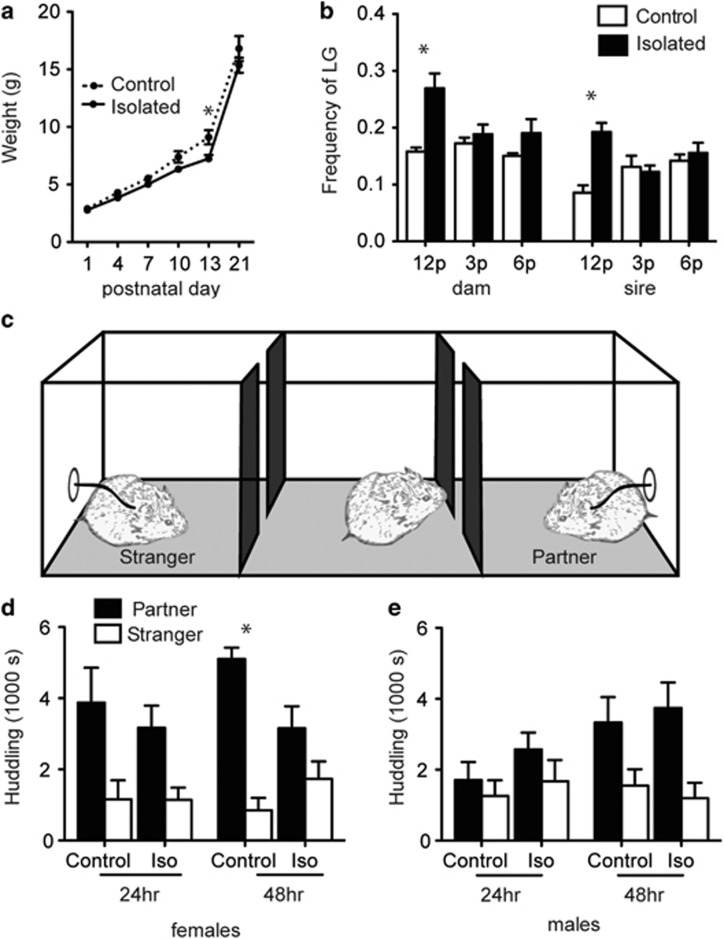
Impact of early isolation on weight, parental care upon reunion and adult behavior. Prairie vole litters were exposed to daily 3h separations from both parents and siblings, or left undisturbed aside from weighing every 3 days. Between PND1–13, weights were averaged across all pups of both sexes within a litter. PND21 weights are averaged across sex. Isolated litters displayed slowed weight gain, and weighed significantly less only on PND13 (**a**). For 1 h after reunion of the litters, both parents displayed heightened licking and grooming in comparison with controls whose pups were not removed from the nest (**b**). Adults were tested for their social preference in the partner preference arena (**c**), reproduced from Barrett and Young.^60^ Only the control females and early-isolated males displayed a significant partner preference after 48 h of cohabitation (**d** and **e**). Asterisk indicates *t*-test, *P*<0.05. PND, postnatal day.

Early-isolated animals displayed slowed weight gain from PND1–13 in comparison with controls (time × rearing condition; F_4,72_=4.78, *P*=0.004; Figure 2a). Weights were normalized by weaning (*P*>0.05).

#### Effect of paradigm on parental care upon reunion

A significant rearing condition by time of day interaction effect was detected in both dams (F_2,20_=8.19, *P*=0.003) and sires (F_2,20_=16.57, *P*<0.001) on the amount of licking and grooming. Licking and grooming from both parents was only enhanced during the 1 h observation upon reunion of isolated pups to the nest (1200 h, dam *P*=0.006, sire, *P*=0.030; Figure 2b), but not at later time points in the day (*P*>0.05). No significant differences in the frequencies of nursing, nest occupancy or non-parental activities were detected (Supplementary Table S1).

#### Open field

Early isolation did not impact any measure of anxiety-like behavior in the open-field test (*P*>0.05, duration in center (s): female, control 6.41±2.62, isolated 12.42±7.82; male, control 12.88±3.99, isolated 14.13±4.24; distance in center (mm): female, control 366.30±156.96, isolated 254.36±81.21; male, control 682.03±191.57, isolated 870.61±247.78).

#### Partner preference

Adults were tested for PP (Figure 2c). After 24 h of cohabitation, there was a main effect of stimulus (F_1,76_=13.71, *P*<0.001) and a stimulus by sex interaction (F_1,76_=4.27, *P*=0.042). Within females, neither rearing group formed a significant PP after 24 h (*P*>0.05, Figure 2d), although there was a main effect of stimulus animal (F_1,36_=12.93, *P*=0.001). No impact of the early isolation on male PP was detected after 24 h of cohabitation (Figure 2e). After 48 h from the time of pairing, there was a main effect of stimulus (F_1,76_=42.37, *P*<0.001) and an interaction between condition, stimulus and sex (F_1,76_=5.44, *P*=0.022). Within females, there was a main effect stimulus animal (F_1,36_=38.38, *P*<0.001), and an interaction effect between condition and stimulus animal (F_1,366_=9.50, *P*=0.004). Control females spent significantly more time with the partner over the stranger (*P*<0.001), but females socially isolated as neonates did not (*P*=0.219). In males after 48 h, there was a main effect of stimulus animal (F_1,40_=12.88, *P*=0.001), but neither group spent significantly more time huddling with the partner after Bonferroni–Holm correction (early isolated, *P*=0.049, control, *P*=0.123). No difference in distance moved (in mm) in the center arena between rearing groups was detected at 48 h (females: control 26 838.92±5885.42, isolated 44 330.99±10 179.22; males: control 32 716.67±4997.72, isolated 42 469.61±9708.13), indicating that PP results were not due to differences in general locomotion.

#### Autoradiography

No significant difference in OTR was detected in any region examined in either naive or behaviorally experienced animals (Supplementary Figure S1).

#### Relationship between NAcc OTR and behavior

**Figure 3:**
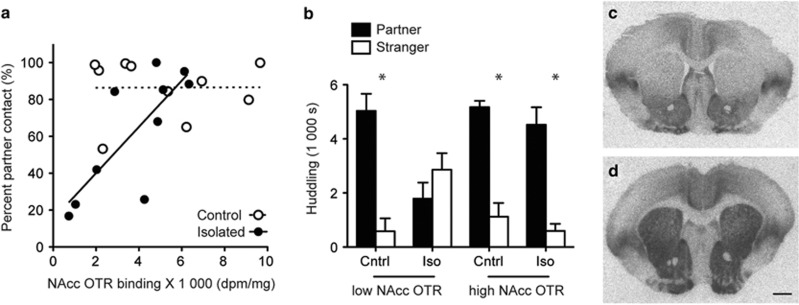
Females with low NAcc OTR are susceptible to early adversity. After 48 h of cohabitation, the percent of time females spent huddling with their partner over total huddling significantly correlated with NAcc OTR binding in the early-isolated (*R*=0.779, *R*^2^=0.607, *P*=0.008), but not control (*R*=0.006, *R*^2^<0.0001, *P*>0.05) females (**a**). Only females with low OTR binding exposed to early isolation did not form a partner preference (**b**). Representative autoradiographs of low (**c**) and high (**d**) OTR NAcc females. Asterisk indicates paired *t*-test, *P*<0.05. Scale bar, 1 mm. NAcc, nucleus accumbens. OTR, oxytocin receptor.

Multiple linear regression revealed a significant interaction effect between rearing condition and NAcc (core and shell combined) OTR binding on percent partner huddling time (ANOVA, F_3,16_=7.50, *P*=0.002, Condition × NAcc OTR, *P*=0.006; Figure 3a), but not with OTR binding in other regions examined (prefrontal cortex, lateral septum, central amygdala, basolateral amygdala, bed nucleus of the stria terminalis; Supplementary Table S2). Percent partner huddling time was used as our primary index of partner preference; however, we also ran *post hoc* multiple linear regressions with partner, stranger, and total huddling time and with open field data as dependent variables (Supplementary Table S2b and S3). The percent of time spent huddling with the partner after 48 h of cohabitation was significantly positively correlated with OTR binding in the NAcc (*R*=0.779, *R*^2^=0.607, *P*=0.008) in the early-isolated group, but not in the control group (*R*=0.006, *R*^2^<0.0001, *P*=0.986; Figure 3a). Females were then divided into either ‘high' or ‘low' OTR NAcc groups using a median split. A three-way ANOVA revealed a significant interaction between stimulus animal and NAcc OTR binding (F_1,32_=9.73, *P*=0.004), stimulus animal and condition (F_1,32_=14.72, *P*=0.001), and a three-way interaction between stimulus animal, rearing condition and NAcc OTR (F_1,32_=13.44, *P*=0.001), suggesting that NAcc OTR binding was related to adult social responses to early isolation. Both low and high OTR expressing control females spent significantly more time with the partner than the stranger (low, *P*=0.015; high, *P*=0.002, Figure 3b). Only early-isolated females in the high accumbal OTR group spent more time with the partner than the stranger (low, *P*=0.417; high *P*=0.010; Figure 3b). Thus, females with high accumbal OTR (Figures 3c and d) were resilient to impairments in PP that resulted from early isolation.

### Experiment 2. Effect of tactile stimulation on immediate-early gene expression in PVN oxytocin and vasopressin neurons

**Figure 4:**
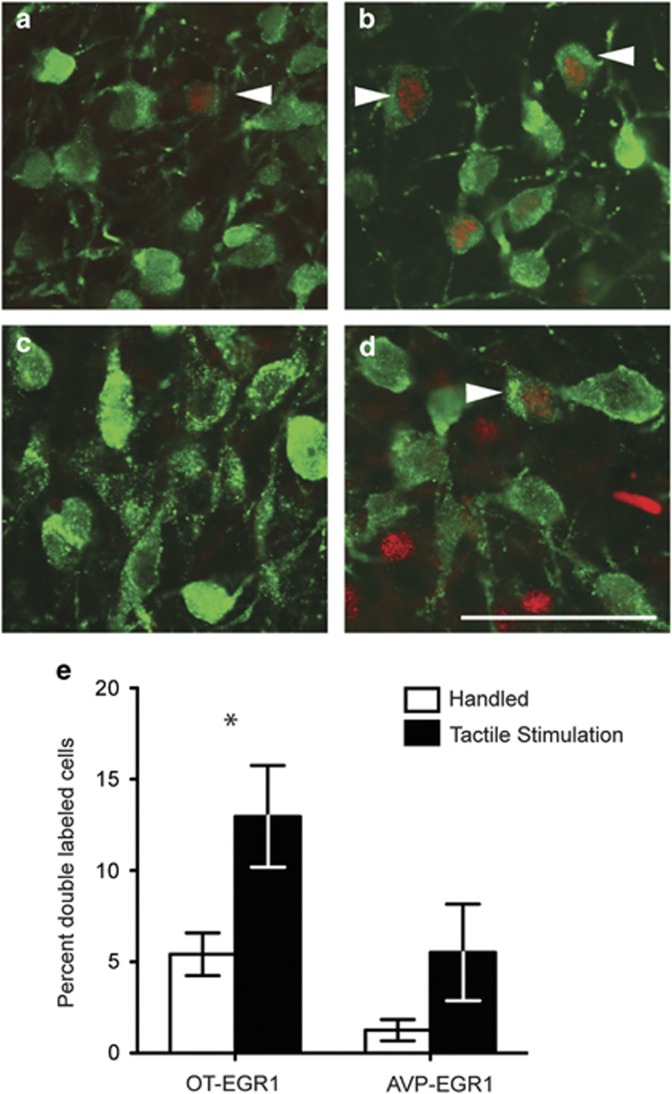
Tactile stimulation activates OT neurons in the PVN. PND6–7 neonates were brushed for 5- min to the anogenital and body region or only handled and killed after 1 h. Representative oxytocin (**a** and **b**) and vasopressin (**c** and **d**) sections in handled (left) and tactile stimulated (right) animals. White arrows indicate cells double labeled for EGR-1 and OT or AVP. Oxytocin cells are green and EGR-1 nuclei are labeled red. Tactile stimulation induced significant EGR-1 activity in oxytocin, but not vasopressin, neurons (**e**). Asterisk indicates Student's *t*-test, *P*<0.05. AVP, vasopressin; EGR-1, early growth response factor-1; OT, oxytocin; PVN, paraventricular nucleus.

Tactile stimulation significantly increased the percentage of PVN OT neurons double labeled for EGR-1 (*P*=0.035) and the number of EGR-1 immunolabeled cells (*P*=0.008). No significant differences in AVP-EGR-1 immunostaining were detected (*P*=0.165; Figures 4a and e). Thus, simulated licking and grooming in a week-old prairie vole significantly activated OT, but not AVP hypothalamic neurons. There was no difference in the raw counts of OT, AVP, EGR-1 in AVP-positive sections or co-labeled cells (Supplementary Table S4). The finding that EGR-1 is increased in the OT analysis, but not the AVP analysis, is likely due to the fact that OT neurons are located at a more anterior portion of the PVN than AVP neurons.

### Experiment 3. MTII effects on buffering against neonatal social isolation

#### Weight

**Figure 5:**
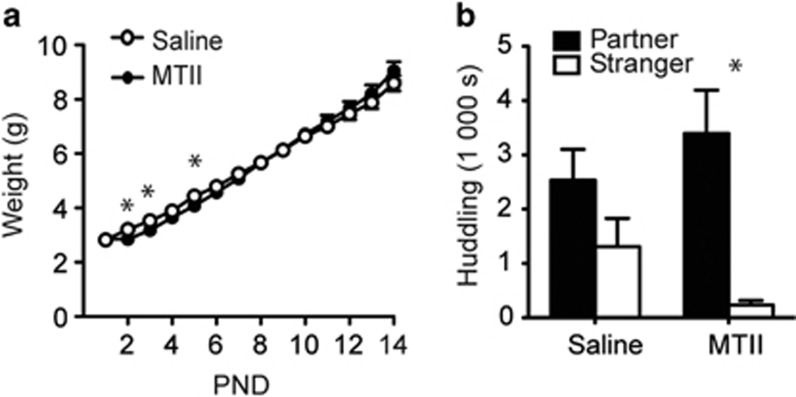
MTII buffered against early isolation in females. Voles were treated with the melanocortin agonist MTII for the first week of the two-week isolation. Females displayed slowed weight gain for the first week of life, but accelerated weight gain when the drug was off-board (**a**). MTII treatment significantly reduced weights on PND2 (*P*=0.012), 3 (*P*=0.032) and 5 (*P*=0.036). Neonatal MTII treatment facilitated female pair bonding after a 6h non-mated cohabitation (**b**). Asterisk indicates *t*-test (**a**) or Wilcoxon test (**b**), *P*<0.05. MTII, melanotan-II; PND, postnatal day.

A repeated measure ANOVA within each sex revealed a significant interaction between drug and PND (F_13,354_=2.77, *P*=0.001) in females (Figure 5a). MTII treatment significantly reduced female weights on PND2, 3, 5.

#### Partner preference

After an abbreviated 6 h non-mated cohabitation, an ANOVA (stimulus animal × treatment) on log-transformed data revealed a main effect of partner (F_1,54_=16.78, *P*<0.001), but a nonsignificant interaction effect (F_1,54_=1.69, *P*=0.199). Planned pairwise Wilcoxon sign rank tests revealed that the isolated females treated with daily MTII spent significantly more time huddling with the partner over the stranger (*P*=0.002; Figure 5b), whereas the saline-injected controls did not (*P*=0.211).

## Discussion

Repeated daily isolations from both siblings and parents over the first 2 weeks of life impaired adult PP in female prairie voles, without impacting open-field behavior. There was no evidence that neonatal isolation altered OTR density in any brain region analyzed. However, NAcc OTR binding significantly correlated with PP behavior in neonatally isolated females, and those with high densities of NAcc OTR were resilient to the impact of neonatal social isolation on later PP. Neonatal isolation stimulated increased licking and grooming from both parents on the pups return to the nest. Tactile stimulation mimicking licking and grooming resulted in increased immediate-early gene EGR-1 immunoreactivity in hypothalamic OT, but not AVP, neurons, suggesting parental nurturing induces OT signaling, which would be intensified in animals with higher OTR density. Finally, MTII, which activates OT neurons in neonates^54^ and adults, and potentiates stimulus-evoked OT release in the adult prairie vole brain,^52^ rescued the isolation-induced impairments in adult bonding. Together, these data suggest that parental nurturing shapes neural systems involved in the formation of social relationships, which may be mediated, in part, by striatal OTR signaling in female prairie voles.

Pair bonding in female voles may be more sensitive to reductions in early care.^61, 62^ Exposure to either neonatal isolations or paternal deprivation alters NAcc dopaminergic signaling in a sexually dimorphic manner in monogamous voles.^45, 62^ Dopamine receptor 2, which promotes social bond formation, is reduced in females, but enhanced in males, exposed to these early insults, potentially suggesting that social attachment is less rewarding for these females. As stress and corticotropin-releasing factor administration promotes pair bonding in male prairie voles,^63, 64^ enhanced stress reactivity in adulthood may override negative effects of reduction in social interactions early in life on male sociality. Furthermore, maternal separations in rats have been reported to enhance adult AVP expression,^39, 65, 66^ which would also serve to promote male bonding. The mechanisms by which male vole sociality is more resilient to early social isolations remain to be determined. However, as control males in this study did not show a PP, we cannot conclusively determine whether neonatal isolation had any impact on social behavior. Our male data are simply inconclusive, whereas the data in females are compelling.

The present study suggests that NAcc OTR density is not significantly affected by early experience in prairie voles, consistent with a previous study where parental nurturing was manipulated.^42^ Indeed, NAcc OTR density appears to be a fixed trait and recent data suggests that variation in NAcc OTR density is associated with a genetic polymorphism in the OTR gene (*Oxtr*).^67^ Although not impacted by experience, striatal OTR density predicted behavioral outcomes of early isolation. NAcc OTR binding significantly correlated with the percent of time females spent huddling with their partner in the early-isolated group, and low expressing females failed to show a PP after 48 h of cohabitation. Non-isolated females displayed no relationship between accumbal OTR and partner huddling and formed a robust PP. Thus, it appears that the low levels of NAcc OTR binding are sufficient to form a PP or that lower OTR signaling can be compensated by other factors, unless the animal experienced neonatal social isolation. This interaction between receptor expression and early experience suggests that oxytocin activity in the NAcc may mediate vulnerability or resilience to adverse life events. However, it should be noted that OTR binding in the entire striatum (NAcc, caudate, putamen, olfactory tubercle) is highly correlated, and our study is not capable of distinguishing which striatal subregions is contributing to this effect. A potential caveat to these interpretations is that, since the offspring were not cross-fostered, parents of high NAcc OTR females may also have high NAcc OTR expression and higher levels of parental care, contributing to the observed resilience. However, this is unlikely given that in our general colony, pups within litters show high variability in OTR owing to genetic heterozygosity in the breeders (King and Young, unpublished data). Another intriguing possibility is that females with high OTR could be eliciting more parental care from their parents, perhaps by emitting increased numbers of ultrasonic vocalizations. The potential caveats should be investigated in future studies.

OT, but not AVP, neurons in the PVN displayed significant immediate-early gene activity in response to a 5 min tactile stimulation in week-old prairie vole neonates. After the 3 h separation, parental care was enhanced upon reunion with the litter, as shown in rats.^68^ It is conceivable that individuals with high OTR sensitivity would experience more OTR signaling from this burst in care and the touch-induced OT release upon reunion. Repeatedly stroking pups with a paint brush alone to mimic licking reduces separation distress^69^ and alleviates behavioral deficits associated with early deprivation in both mice^9^ and voles.^10^ In addition to tactile stimulation, other sensory cues (for example, auditory, temperature) are likely important components of parental care, and tactile stimulation alone may not precisely recapitulate the effects of parental nurturing on hypothalamic OT neurons. Nonetheless, developmental OTR signaling may strengthen circuitry involved in the development of social bonding. Indeed, long-term developmental elevation of NAcc OTR from weaning age, but not acutely in adulthood, increases adult alloparental care towards novel pups,^29, 30^ suggesting an important developmental role for striatal OTR signaling. In rodents, neonatal OT regulates maternal distress calls^70, 71, 72^ and the development of a preference for maternal odors.^73, 74^ Postnatal OT also mediates the GABA switch from excitatory to inhibitory, an effect disrupted in mouse models of autism,^75, 76^ and sensory experience-dependent cortical plasticity.^77^ Whether OT can mediate plasticity in non-cortical regions, such as limbic regions underlying social behavior, is an exciting area of future research. The mechanisms by which resilience is mediated by accumbal oxytocin signaling, such as through interactions with accumbal dopaminergic^78^ or serotonergic^79^ pathways, warrants further investigation.

Alpha-melanocyte stimulating hormone robustly stimulates somatodendritic release of OT by activating MC4Rs in rat hypothalamic slices.^53^ We have shown that in adults the MC3/4R agonist MTII stimulates EGR-1 immunoreactivity in OT neurons and potentiates hypertonic saline-induced OT release into the NAcc.^52^ Given the relationship between NAcc OTR and resilience to neonatal social isolation, we sought to determine whether a pharmacological manipulation known to activate the OT system could rescue social impairments induced by isolation. We found that MTII administration for the first week of life buffered against the early isolation-induced PP impairments in females. This is consistent with previous results in socially unmanipulated pups where daily neonatal MTII enhances adult PP formation in female, but not male, prairie voles.^54^ Future experiments will assess whether other developmental windows exist in which MTII buffer against the early social stress.

Early endogenous or pharmacological activation of the OT system has life-long impacts on social behavior and neuroendocrine function.^80^ A single peripheral dose of OT to female prairie voles at birth impacts a number of social behavioral measures including maternal care and PP.^81, 82^ Early OT facilitates the establishment of a PP in a dose-dependent fashion. These findings parallel the effect of neonatal MTII administration on adult bond formation and support an OT-based mechanism of action. Moreover, early administration of OT or an MC4R agonist rescues social deficits in mice with autistic phenotypes.^83, 84^ Although our results are in line with an OT-dependent mechanism, the MC4R also interacts with the dopamine system in rats^85^ and activates corticotropin-releasing factor and AVP neurons in prairie vole pups,^54^ and thus, our study cannot be construed as proof that MCR effects on behavior are due to effects on the OT system. Side effects of melanocortin activation, such as increased blood pressure and heart rate, an enhancement of sexual activity and reduced appetite, must be considered before pursuing this avenue as a therapeutic.^86, 87^

### Human relevance

A growing body of literature has demonstrated the importance of OT signaling in mediating social behavior, outcomes of early adversity, and parent–infant interactions in humans.^8,88^ Single-nucleotide polymorphisms of the human *OXTR* are associated with socioemotional behavior in humans, including variability in parental sensitivity,^89^ empathy and stress reactivity,^90^ affect and self-esteem,^91, 92^ social recognition^93^ and psychopathologies including depression, anxiety^94^ and autism.^95, 96, 97, 98^ Early adverse experiences are associated with reduced cerebrospinal fluid OT concentrations in adult humans and rhesus macaques^99, 100^ as well as lowered urinary OT in abused children.^8, 101^ Recently, it has been suggested that *OXTR* polymorphisms interact with early-life adversity, including maternal depression, childhood maltreatment and war exposure, to enhance the risk for exhibiting psychological symptoms,^46, 47, 48, 49, 102, 103^ and female adolescents may be particularly susceptible,^48, 102^ paralleling our finding that female voles were more responsive to early isolations. *OXTR* polymorphisms also associate with resilient functioning in response to positive early environments.^104, 105^ Thus, the *OXTR* gene can interact with early experience to impact adult social behavior. Although the first week of life in rodents may be equivalent to the late prenatal period in humans,^106^ OT may have a similar role across mammalian species in integrating early sensory and social information. Indeed, OT release is coordinated between parents and infants during social interactions^19^ and *OXTR* polymorphisms have been linked to the security of infant attachment,^107^ suggesting that OT early in life may regulate early social interactions in humans in a similar manner to voles. A 15 min massage increases plasma OT levels in adults^108^ and parent–infant interactions increase salivary OT in infants.^19, 20^ OT increases the perceived pleasantness of social touch and enhances functional magnetic resonance imaging responses in the insula, precuneus, orbitofrontal and pregenual cortices.^109^ The cumulative presence of OT and vasopressin risk alleles increase vulnerability to developing posttraumatic stress disorder in war-exposed children, and maternal support improves psychological outcomes.^103^ These and our results suggest that enhancing early OT release in cases of adversity or neglect in humans may be a viable preventative treatment for a number of psychiatric conditions.

## Conclusion

To our knowledge, these results are the first to report that variation in neural OTR density is associated with variation in responses to early adversity, and suggest that hypothalamic OT may mediate responses to early touch in prairie voles. This study is the first to establish a relationship between individual variation in neuropeptide receptor expression in the brain and susceptibility/resilience to early-life social experience/neglect. Child abuse, parental neglect and preterm infancy all involve disruptions in socioemotional development and health, thus development of interventions or therapeutics is critical. Drugs that stimulate OT release may be viable candidates for improving social cognition in psychiatric conditions associated with abnormal social functioning.^110, 111^ Our data provide intriguing evidence that pharmacological potentiation of the OT system may augment social experiences and have potential beneficial effects on the development of neural pathways involved in social engagement. However, it should be noted that other studies in prairie voles have found that daily intranasal OT in adolescence actually reduced PP formation in adult male prairie voles.^112^ Therefore, more research is needed to understand the potential beneficial, as well as detrimental, impacts of manipulating the OT system developmentally.^113^ Nonetheless, early manipulation of the OT system through the administration of MCR agonists may have the ability to evoke long-term changes in social behavior.

## Notes

LJY has applied for a patent (US20120108510—Methods of improving behavioral therapies) for combining MCR agonists with behavioral therapies to enhance social cognition in psychiatric disorders. The remaining authors declare no conflict of interest.
