# Supplementary material for: The oxytocin system promotes resilience to the effects of neonatal isolation on adult social attachment in female prairie voles
Source: Transl Psychiatry. 2015 Jul 21;5(7):e606–. doi: 10.1038/tp.2015.73 (PMC5068726; doi:10.1038/tp.2015.73)
Supplement: Supplementary Information [file tp201573x1.pdf]

Supplemental results

Figure S1. OTR autoradiography in females. No difference in OTR binding between control and neonatally isolated females were detected. Data is combined across animals that were either paired with a male partner or unpaired (and naïve to behavioral testing).

Table S1. Behavior of parents upon reunion of pups at 12PM from PND1-14. Descriptive statistics and t-tests results between control and isolated litters are listed for the 1 hr observation at 12PM directly after reunion. Means are presented as the ratio of total observations (15 observations per hour, 3 hours per day, PND1-14). Uncorrected p-values are presented. Licking and grooming data is presented in Figure 1.

Behavior	Parent	Control			Isolated			P-value
		Mean	SE	N	Mean	SE	N	
On nest	Female	0.809	0.044	5	0.898	0.020	7	0.067
On nest	Male	0.904	0.016	5	0.924	0.019	7	0.447
Arched-back nursing	Female	0.721	0.054	5	0.829	0.025	7	0.075
Blanket nursing	Female	0.001	0.001	5	0.000	0.000	7	0.255
Side nursing	Female	0.083	0.009	5	0.054	0.015	7	0.166
Nest building	Female	0.038	0.017	5	0.024	0.010	7	0.470
Nest building	Male	0.021	0.006	5	0.024	0.008	7	0.758
Carrying	Female	0.007	0.002	5	0.022	0.006	7	0.074
Carrying	Male	0.001	0.001	5	0.016	0.005	7	0.018
Climbing	Female	0.009	0.004	5	0.002	0.001	7	0.098
Climbing	Male	0.006	0.002	5	0.004	0.004	7	0.763
Drinking	Female	0.013	0.005	5	0.018	0.002	7	0.390
Drinking	Male	0.007	0.004	5	0.003	0.001	7	0.273
Digging	Female	0.013	0.005	5	0.003	0.001	7	0.060
Digging	Male	0.011	0.004	5	0.004	0.002	7	0.106
Eating	Female	0.119	0.030	5	0.069	0.011	7	0.110
Eating	Male	0.052	0.009	5	0.046	0.010	7	0.663
Grooming mate	Female	0.000	0.000	5	0.001	0.001	7	0.424
Grooming mate	Male	0.004	0.002	5	0.006	0.002	7	0.503
Grooming self	Female	0.016	0.005	5	0.013	0.003	7	0.583
Grooming self	Male	0.043	0.005	5	0.042	0.009	7	0.953
Resting	Female	0.560	0.050	5	0.571	0.025	7	0.837
Resting	Male	0.716	0.028	5	0.612	0.029	7	0.030
Wandering	Female	0.035	0.008	5	0.015	0.005	7	0.059
Wandering	Male	0.016	0.007	5	0.024	0.005	7	0.366

Table S2. Interaction between rearing condition, OTR binding, and partner preference in females. Results from multiple linear regressions performed separately for OTR binding in each brain region are listed (ANOVA, Unstandardized coefficients (B, Std Error) for main effects (condition, binding) and condition X binding interaction effects). Predictors were rearing condition, OTR binding, and a condition by OTR binding interaction term. The percent time huddling with the partner was the primary dependent variable, and is an index of partner preference (A). We also analyzed partner huddling time, and total huddling time with partner and stranger as dependent variables *post hoc* (B). Asterisks indicate statistically significant interaction effects with Bonferroni-Holm correction for multiple comparisons (corrected α = 0.008 for first step-wise comparison).

A.

	ANOVA		Condition			Binding			Interaction		
Region	F (3,16)	P value	B	SE	P value	B	SE	P value	B	SE	P value
Percent time huddling with partner over total huddling (partner + stranger) time											
NAcc	7.50	.002	-71.31	19.01	.002	-.012	.006	.04	.013	.004	.006*
BLA	3.42	.043	-82.67	34.03	.027	-.016	.011	.163	.017	.009	.069
BNST	1.83	.182	-55.69	30.69	.088	-.014	.013	.320	.013	.011	.263
CeA	3.00	.062	-54.74	25.68	.049	-.006	.007	.391	.007	.005	.166
LS	1.51	.250	-4.73	27.19	.864	.019	.024	.432	-.012	.015	.458
PFC	2.27	.119	-89.12	44.55	.063	-.018	.014	.221	.015	.010	.149

B.

	ANOVA		Condition			Binding			Interaction		
Region	F (3,16)	P value	B	SE	P value	B	SE	P value	B	SE	P value
Total time huddling with partner											
NAcc	6.30	.005	-4145	1299	.006	-.548	.382	.171	.589	.269	.044
BLA	8.09	.002	-6157	1788	.003	-1.06	.568	.081	1.21	.448	.016
BNST	3.08	.058	-3402	1843	.084	-.517	.803	.528	.626	.676	.368
CeA	4.14	.024	-3250	1567	.055	-.195	.445	.667	.316	.303	.313
LS	2.62	.086	-960.5	1641	.567	1.09	1.44	.461	-.602	.927	.525
PFC	3.49	.040	-5281	2692	.067	-.776	.868	.385	.771	.608	.223
Total time huddling with stranger											
NAcc	5.99	.006	3427	999.4	.003	.682	.294	.034	-.661	.207	.006*
BLA	2.68	.082	4060	1758	.035	.865	.558	.140	-.892	.441	.060
BNST	1.50	.252	3010	1552	.070	.936	.676	.185	-.859	.569	.151
CeA	2.27	.119	2561	1327	.072	.356	.377	.359	-.388	.257	.150
LS	.993	.421	-279.1	1397	.844	-1.20	1.23	.344	.714	.790	.379
PFC	2.11	.139	5083	2225	.036	1.23	.718	.106	-.980	.503	.069
Total huddling time											
NAcc	1.97	.158	-717.4	980.3	.475	.134	.288	.647	-.072	.203	.727
BLA	3.08	.057	-2097	1351	.140	-.193	.429	.658	.316	.339	.365
BNST	2.50	.097	-391.7	1142	.736	.419	.498	.412	-.233	.419	.586
CeA	2.07	.144	-689.2	1060	.525	.161	.301	.600	-.072	.205	.728
LS	1.88	.173	-1239	1030	.247	-.107	.906	.907	.112	.582	.850
PFC	2.36	.110	-198.1	1724	.910	.453	.556	.427	-.209	.389	.598

Table S3. Interaction between rearing condition, OTR binding, and open field data in females. Results from multiple linear regressions performed separately for OTR binding in each brain region are listed (ANOVA, Unstandardized coefficients (B, Std Error) for main effects (condition, binding) and condition X binding interaction effects). Predictors were rearing condition, OTR binding, and a condition by OTR binding interaction term. Dependent variables were duration and distance in the center of the open field.

Region	ANOVA		Condition			Binding			Interaction		
	F (3,16)	P value	B	SE	P value	B	SE	P value	B	SE	P value
Duration in center of open field											
NAcc	1.28	.314	-14.48	17.32	.415	-.005	.005	.305	.005	.004	.156
BLA	.350	.790	17.37	27.63	.538	.002	.009	.781	-.003	.007	.632
BNST	.952	.439	32.18	21.44	.153	.011	.009	.274	-.011	.008	.187
CeA	.203	.893	-.650	20.61	.975	-.002	.006	.750	.001	.004	.723
LS	.742	.543	17.67	18.88	.363	.006	.017	.728	-.008	.011	.471
PFC	1.30	.308	-46.00	31.21	.160	-.014	.010	.179	.012	.007	.106
Distance in center of open field											
NAcc	.232	.873	-316.4	403.4	.444	-.065	.119	.593	.048	.084	.573
BLA	.437	.730	.094	585.9	1.00	-.003	.186	.986	-.042	.147	.776
BNST	.345	.794	135.9	481.8	.781	.088	.210	.680	-.107	.177	.554
CeA	.235	.871	-269.2	439.0	.548	-.061	.125	.629	.031	.085	.722
LS	.195	.898	-199.0	422.8	.644	-.039	.372	.917	.059	.239	.807
PFC	.710	.560	-909.3	698.6	.211	-.295	.225	.209	.188	.158	.252

Table S4. Impact of tactile stimulation on OT and AVP neuron activity in the PVN. Descriptive statistics of OT, AVP, EGR1, and co-labeled cells, as well as significant differences between handling and tactile stimulation groups are listed.

	Handling			Tactile Stimulation			T-test
	Mean	SE	N	Mean	SE	N	P value
Oxtocin-EGR1							
Total # OT	479.71	64.94	7	467.00	32.20	8	.858
Total # EGR1	322.71	47.84	7	534.37	46.73	8	.008*
Total # OT-EGR1 co-labeled	28.71	7.76	7	56.25	10.17	8	.056
Percent EGR1-OT co-labeled	5.42%	1.17	7	12.96%	2.80	8	.035*
Vasopressin-EGR1							
Total # AVP	232.71	24.88	7	217.13	25.50	8	.671
Total # EGR1	256.14	41.89	7	341.25	46.62	8	.203
Total # AVP-EGR1 co-labeled	2.71	1.29	7	13.38	7.44	8	.210
Percent EGR1-AVP co-labeled	1.26%	.58	7	5.52%	2.65	8	.165
