# Supplementary figures and images for: The oxytocin system promotes resilience to the effects of neonatal isolation on adult social attachment in female prairie voles
Source: Transl Psychiatry. 2015 Jul 21;5(7):e606–. doi: 10.1038/tp.2015.73 (PMC5068726; doi:10.1038/tp.2015.73)

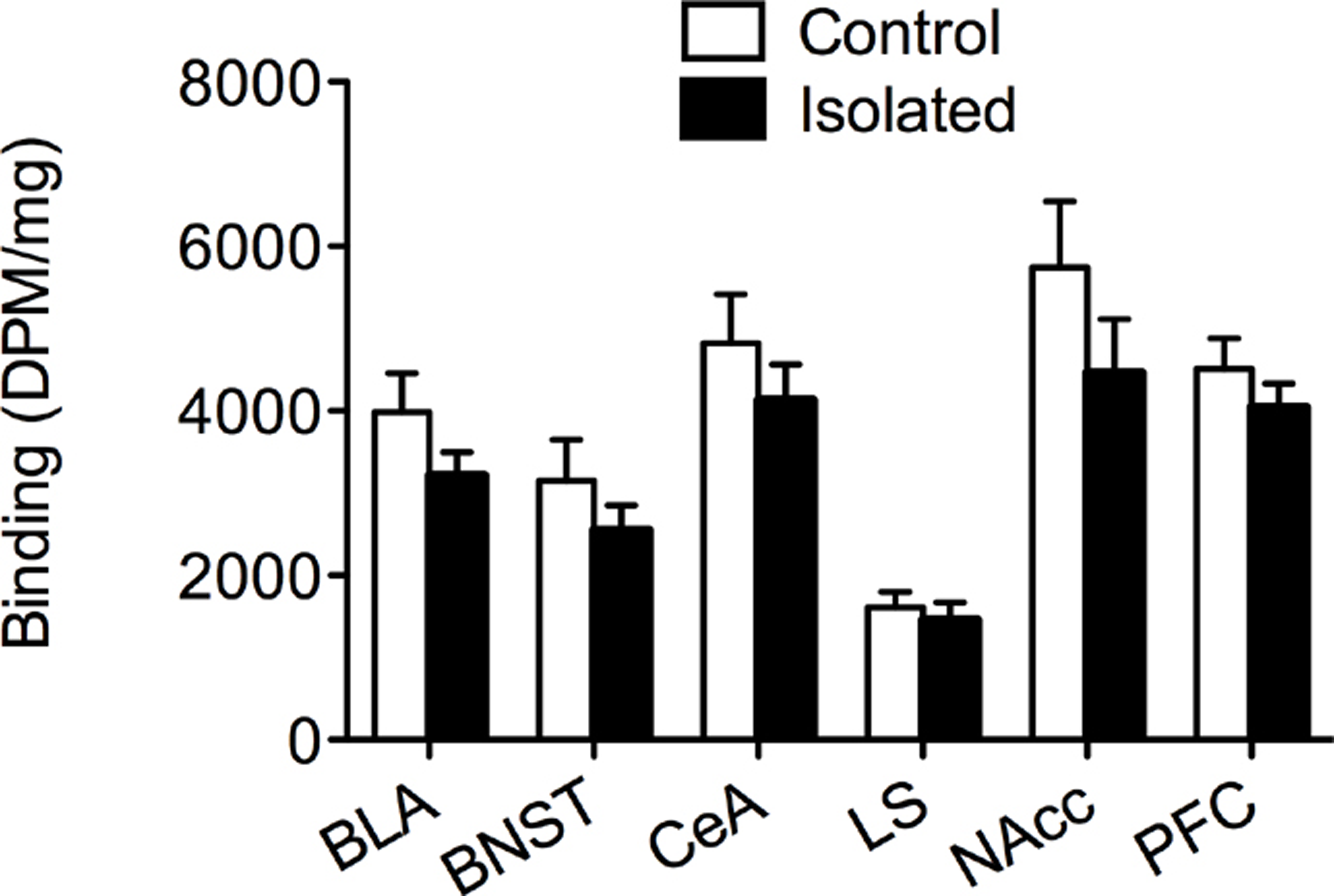

Supplement: Supplementary Figure 1 [file tp201573x2.tif]
